# Elevated type-17 cytokines are present in axial spondyloarthritis stool

**DOI:** 10.1093/discim/kyae005

**Published:** 2024-05-04

**Authors:** India Brough, Kelsey Thompson, Ciara Latore, Frank Penkava, Chelsea Regan, Claire Pearson, Hui Shi, Anna Ridley, Davide Simone, Lilian Lam, Samuel Bullers, Caroline Moussa, Rachel Feeney, Inflammatory Arthritis Microbiome Consortium (IAMC) Investigators Group, Mohammed H Al-Mossawi, Fiona Powrie, Stephen Young, Curtis Huttenhower, Paul Bowness

**Affiliations:** NDORMS, Oxford University, Oxford, UK; Chan School of Public Health, Harvard University, Boston, MA, USA; NDORMS, Oxford University, Oxford, UK; NDORMS, Oxford University, Oxford, UK; Institute of Inflammation and Ageing, University of Birmingham, Birmingham, UK; NDORMS, Oxford University, Oxford, UK; NDORMS, Oxford University, Oxford, UK; NDORMS, Oxford University, Oxford, UK; NDORMS, Oxford University, Oxford, UK; NDORMS, Oxford University, Oxford, UK; NDORMS, Oxford University, Oxford, UK; NDORMS, Oxford University, Oxford, UK; NDORMS, Oxford University, Oxford, UK; NDORMS, Oxford University, Oxford, UK; NDORMS, Oxford University, Oxford, UK; Institute of Inflammation and Ageing, University of Birmingham, Birmingham, UK; Chan School of Public Health, Harvard University, Boston, MA, USA; NDORMS, Oxford University, Oxford, UK

**Keywords:** AxSpA, Microbiome, Amino acids, Th17, IL-23, IL-17

## Abstract

Axial spondyloarthritis (axSpA) is characterized by type-17 immune-driven joint inflammation, and intestinal inflammation is present in around 70% of patients. In this study, we asked whether axSpA stool contained Th17-associated cytokines and whether this related to systemic Th17 activation. We measured stool cytokine and calprotectin levels by ELISA and found that patients with axSpA have increased stool IL-17A, IL-23, GM-CSF, and calprotectin. We further identified increased levels of circulating IL-17A+ and IL-17F+ T-helper cell lymphocytes in patients with axSpA compared to healthy donors. We finally assessed stool metabolites by unbiased nuclear magnetic resonance spectroscopy and found that multiple stool amino acids were negatively correlated with stool IL-23 concentrations. These data provide evidence of type-17 immunity in the intestinal lumen, and suggest its association with microbial metabolism in the intestine.

## Graphical Abstract

**Graphical Abstract:**
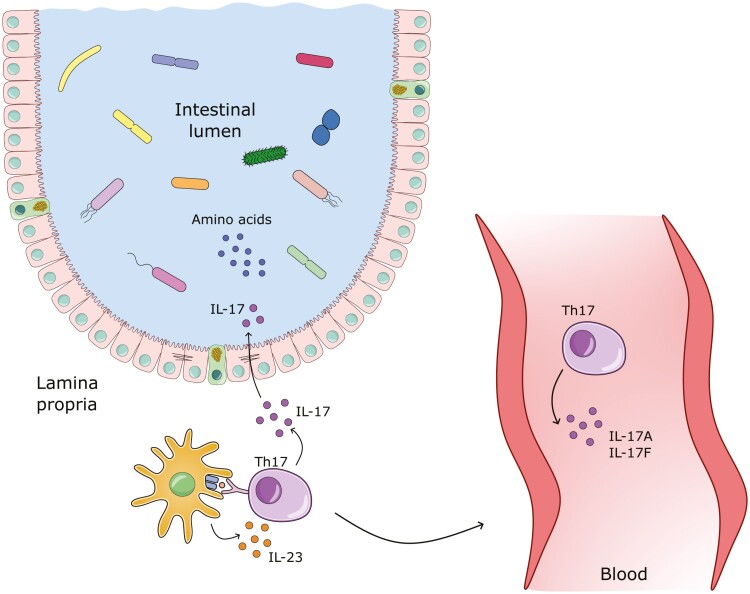

## Introduction

Axial spondyloarthritis (axSpA) is a common form of inflammatory arthritis typically characterized by inflammation of the axial skeleton and large joints [1]. In addition to joint inflammation, subclinical intestinal inflammation is present in roughly 70% of patients [2]. The pathogenesis of axSpA is unclear, however, HLA-B27 is the strongest genetic factor and is present in ~90% of patients [3] Genome-wide association studies (GWAS) have identified common genes associated with inflammatory bowel disease (IBD) and axSpA, including IL-23R and other type 17-associated genes, indicating a gut–joint axis in axSpA [4]. The concordance rate for axSpA in monozygotic twins is 63%, suggesting an additional role for environmental factors in driving axSpA development [5]. For instance, studies in animal models of axSpA demonstrate a requirement for the intestinal microbiome to support joint inflammation [6, 7]. Furthermore, patients with axSpA display altered intestinal microbiome composition compared to healthy individuals [8–10]. Intestinal bacteria can modulate the host immune system through the secretion of metabolites [11]. Indeed, oral administration of the metabolite butyrate can attenuate arthritis in mice, suggesting an important role for intestinal metabolites in arthritis pathology [12]. We, therefore, sought evidence of an altered metabolic landscape in the stool of axSpA through unbiased NMR spectroscopy and asked whether this was associated with a type-17 response in axSpA stool by utilizing stool cytokine quantification [13]. Previous studies have demonstrated enrichment in peripheral IL-17A-producing lymphocytes in patients with axSpA, and a question arises as to whether this peripheral Th17 response is driven by the intestinal microenvironment [14]. To answer this question, we additionally performed intracellular T cell cytokine flow cytometry on peripheral blood mononuclear cells (PBMCs) and ran correlation analyses with stool proteins in patients with axSpA.

## Methods and materials

### Patient and control recruitment

A total of 53 axSpA patients meeting the Assessment of Spondyloarthritis International Society (ASAS) classification criteria [15] were recruited at the Oxford University Hospitals National Health Service Foundation Trust, and fresh-frozen (*n* = 30) and 100% ethanol-fixed stool (*n* = 53), and peripheral blood (*n* = 46) samples were obtained. Patients who were diagnosed with cancer, presenting with active IBD, pregnant, or under the age of 18 at the time of recruitment were excluded. Ninety-one sex and age-matched healthy controls were recruited, and 83 of these (40 HLA-B27 positive and 39 HLA-B27 negative) were recruited through the Oxford BioBank. Patient and control demographics are outlined in Supplementary Table 1. Informed written consent was obtained (Ethics 06/Q1606/139, National Health Service, Health Research Authority, South Central—Oxford C Research Ethics Committee).

### Sample collection

Blood was collected from patients at clinical visits and from healthy controls upon their visit to the Oxford BioBank. The serum was extracted by centrifugation at 1400 x *g* (av) for 15 min and stored at –80°C.

Participants were provided with home stool-collection kits and stool samples were collected using a FecesCatcher (Tag Hemi) and a stool container (Starstedt). Ethanol-fixed stool samples were placed into 5 ml 100% ethanol (Sigma–Aldrich) and posted to the Kennedy Institute of Rheumatology by next-day Royal Mail delivery. Fresh stool samples were collected and placed into ice packs within disposable Styrofoam containers and returned in person on the day of stool collection. Samples were frozen at –80°C within 30 min upon arrival. A Biopulverizer (Stratech) was used to pulverize fresh-frozen stool whilst cooled in liquid nitrogen. All assays were performed on pulverized aliquots.

### ELISA

One hundred milligrams of fresh-frozen stool was added to 1 ml of cOmplete ULTRA tablets EDTA-free (Roche) using screw cap microcentrifuge tubes (ThermoFisher Scientific) and a TissueLyser II (Qiagen) was used for homogenization at 30 Hz for 10 min, followed by centrifugation (10 000 × *g* (av) for 3 min at 4°C). 70 *μ*m cell strainers (Fisher Scientific) were used to filter supernatants which were then stored at –80°C until ELISA. Levels of proteins (Supplementary Table 2) were assessed in the stool supernatant by ELISA according to the manufacturer’s instructions, incubating samples and standards at 4°C overnight. Levels of serum LBP, s-CD14, and iFABP were quantified by ELISA according to the manufacturer’s instructions (R&D Systems).

### NMR metabolomics

Ethanol-fixed stool samples were dried using a vacuum (18–24 h Speedivac) to remove ethanol until the weight became constant. Tubes were weighed and two 7 mm stainless steel balls (Qiagen) and water (2 ml/g solid material) were added. Tubes were then vigorously shaken in a bead mill (low-setting Qiagen Tissuelyzer II) for 5 min to homogenize the dried stool. Samples were centrifuged (13 000 × *g* (av) 15 min 4°C) and supernatants recentrifuged (13 000 × *g* (av) for 30 min at 4°C). The supernatant was then diluted in a 1:3 ratio with NMR buffer containing 1.6 mM difluorotrimethylsilylmethylphosphonic acid (DFTMP) (Manchester Organics), 400 mm phosphate, 40% D2O, 0.4% azide and 2 mm 3-(trimethylsilyl)-1-propanesulfonic acid-d6 sodium salt (DSS-d6) (Merck). Sixty microlitres were then added to glass champagne vials (Cole-Parmer) and stored at –80°C until analysis. Upon thawing, samples were transferred to 1.7 mm NMR tubes (Bruker Biospin) using an Anachem Autosampler. Tubes were capped and wiped with dust-free paper, and one-dimensional 1H spectra were acquired at 300 K using a standard 1D-1H-Nuclear Overhauser Effect spectroscopy (NOESY) pulse sequence with water saturation using pre-sat in a Bruker AVANCE II 600 MHz NMR spectrometer (Bruker Corp) equipped with a 1.7 mm cryoprobe. A 12 ppm spectral width was set and scans were repeated 128 times. Samples were loaded into racks and kept at 6°C in the SampleJet sample handling device until processed. Unbiased NMR spectroscopy was completed by using Chenomx NMR Suite v8.6 to read the spectra and each was manually compared with the Chenomx 600 Mhz library v11 of NMR spectra containing 336 compounds to annotate metabolites.

Associations of NMR spectroscopy with the patient’s immunophenotypes were analysed in R version 4.1.2 [16], using R Studio. Due to our stool collection methods, which used ethanol to fix the microbial DNA in the samples, we decided to remove ethanol from our metabolomics analysis. Processed NMR data was median normalized using the limma:normalizeMedianValues function [17]. Per-feature models were then constructed using defaults in MaAsLin 2 v1.8.0 [18]. Data was log-transformed and a linear model for each metabolite was constructed adjusting for the experimental NMR batch using the formula: metabolite ~ IL-23 (stool) + MBX batch.

### Intracellular flow cytometry staining

PBMCs were isolated and stimulated as previously described [14]. Cells were stained for surface markers (Supplementary Table 3) for 20 min on ice, and permeabilized using Cytofix/Cytoperm fixation and permeabilization solution (BD Biosciences). Intracellular cytokines were then stained for 30 min at room temperature. Flow cytometry was performed on a BD LSR II flow cytometer calibrated with calibration and tracking beads (BD Biosciences). Data was analysed using FlowJo^™^ v10 software (BD Biosciences) and the batch effect was analysed and ruled out. iMFI was calculated by multiplying the frequency of CD4+ T cells positive for cytokine with MFI of the cytokine production from CD4 + T cells.

### General data analysis

ELISA and flow cytometry data were processed in R version 4.2.1 [19] or GraphPad Prism version 9.2. Benjamini–Hochberg approach was used to correct for multiple comparisons.

## Results

### Stool calprotectin and Th17 cytokines are elevated in AxSpA

**Figure 1:**
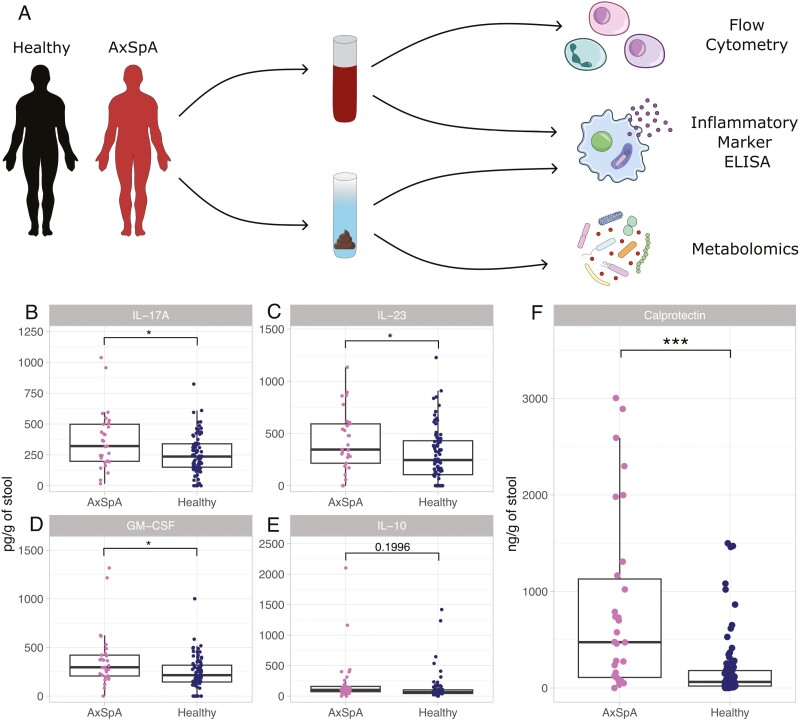
stool calprotectin and Th17 cytokines are elevated in AxSpA. ELISA was completed on fresh stool obtained from 40 patients with axSpA and 85 healthy controls to quantify cytokine levels. (**A**) Sample collection flow chart, (**B**) stool IL-17A, (**C**) stool IL-23, (**D**) Stool GM-CSF, (**E**) Stool IL-10, (**F**) Stool calprotectin (ng/g of stool). Cytokine levels are denoted as pg/g of stool. ***P < 0.001, *P < 0.05. Unpaired student’s *t*-test with Welch’s correction and Benjamini–Hochberg correction for multiple testing. Boxplots denote the median + interquartile range and individual data points are shown.

We first studied fresh stool samples from 40 axSpA patients and 85 age and sex-matched healthy donors (Fig. 1A). We quantified cytokine levels in stool supernatant using ELISA and found that stool IL-17A, IL-23, and GM-CSF were significantly increased in axSpA compared to healthy donors (Fig. 1B–D). No significant difference was identified in stool IL-10 between axSpA patients and healthy donors (Fig. 1E). To determine whether neutrophil-associated molecules indicative of subclinical intestinal inflammation are altered in axSpA, we measured calprotectin (S100A8/9) levels in stool samples. Stool calprotectin was significantly increased in axSpA patients compared to healthy donors (Fig. 1F).

We sought to identify whether patients with high levels of stool cytokines were consistent across the various cytokines tested. Indeed, IL-17A, GM-CSF, and IL-23 all strongly correlated with one another in axSpA and healthy control stool (Supplementary Fig. 1A–C). We found that stool IL-10 did not significantly correlate with stool IL-17A or stool GM-CSF in patients with AxSpA, though did weakly correlate with stool GM-CSF when healthy controls were added (Supplementary Fig. 1D and E). Stool IL-10 significantly correlated with stool IL-23 in both axSpA patients and healthy controls (Supplementary Fig. 1F). Overall, patients with high levels of stool type 17-associated cytokines IL-17A, IL-23, and GM-CSF are consistent across these cytokines but not strongly with IL-10.

Patients with active IBD were excluded from this study, however, patients with inactive IBD were not. Therefore, we sought to identify whether elevated stool cytokine and calprotectin levels were associated with IBD and found that axSpA stool cytokine levels were not significantly associated with IBD status (Supplementary Fig. 2C–E). Furthermore, we found that several axSpA patients with IBD had high levels of calprotectin, though stool calprotectin was not significantly elevated in patients with IBD compared to those without an IBD diagnosis (Supplementary Fig. 2B). Stool calprotectin was still significantly elevated in axSpA patients without IBD, compared to healthy controls (Supplementary Fig. 2A).

Stool calprotectin positively correlated with stool IL-23 in the entire study cohort of axSpA patients and healthy donors, but not if axSpA patients were studied alone (Supplementary Fig. 3). Measures of disease activity and disease duration were not significantly associated with stool cytokine levels or any other immune parameters studied (Supplementary Fig. 4). Non-steroidal anti-inflammatory drug (NSAID) usage has been associated with elevated stool calprotectin [20], however, neither NSAID nor biologic usage significantly impacted stool calprotectin or stool cytokine levels (Supplementary Fig. 5).

We also quantified serum levels of soluble (s)-CD14, a molecule released by innate immune cells following bacterial LPS activation, LPS binding protein (LBP), and intestinal fatty acid binding protein (I-FABP), in serum. We found no significant differences in these markers of intestinal permeability between patients with axSpA and healthy controls (Supplementary Fig. 6).

### Stool amino acid levels negatively correlate with stool IL-23

**Figure 2:**
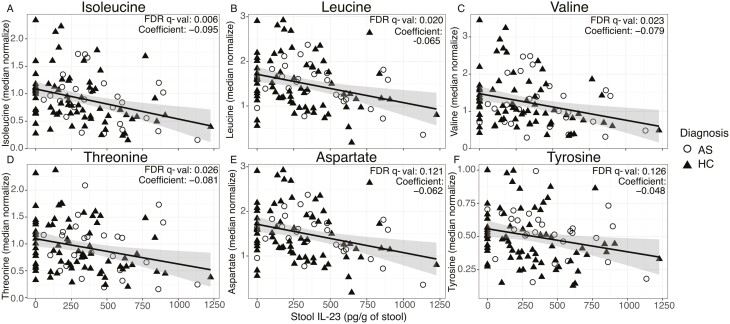
stool amino acid levels negatively correlate with stool IL-23. NMR spectroscopy was completed on ethanol-fixed stool samples from patients with axSpA and healthy controls. Association analyses were performed to identify stool metabolites that are associated with immune parameters. Stool IL-23 was negatively associated with several amino acids. (**A**) Isoleucine, (**B**) leucine, (**C**) valine, (**D**) threonine, (**E**) aspartate, (**F**) tyrosine. Linear modelling was completed using the formula: metabolite ~ IL-23 (stool) + MBX batch. Individual points are shown.

To identify stool metabolites associated with axSpA, we completed unbiased metabolomics on stool samples from 62 axSpA patients and 27 sex and age-matched healthy donors. Association analysis across all annotated metabolites identified multiple essential and non-essential amino acids negatively correlating with stool IL-23 concentration. Indeed, isoleucine, leucine, valine, threonine, aspartate, and tyrosine concentrations were all negatively correlated with stool IL-23 levels (Fig. 2).

### AxSpA patients have increased frequencies of circulating Th17 cells which do not correlate with stool cytokine levels

**Figure 3:**
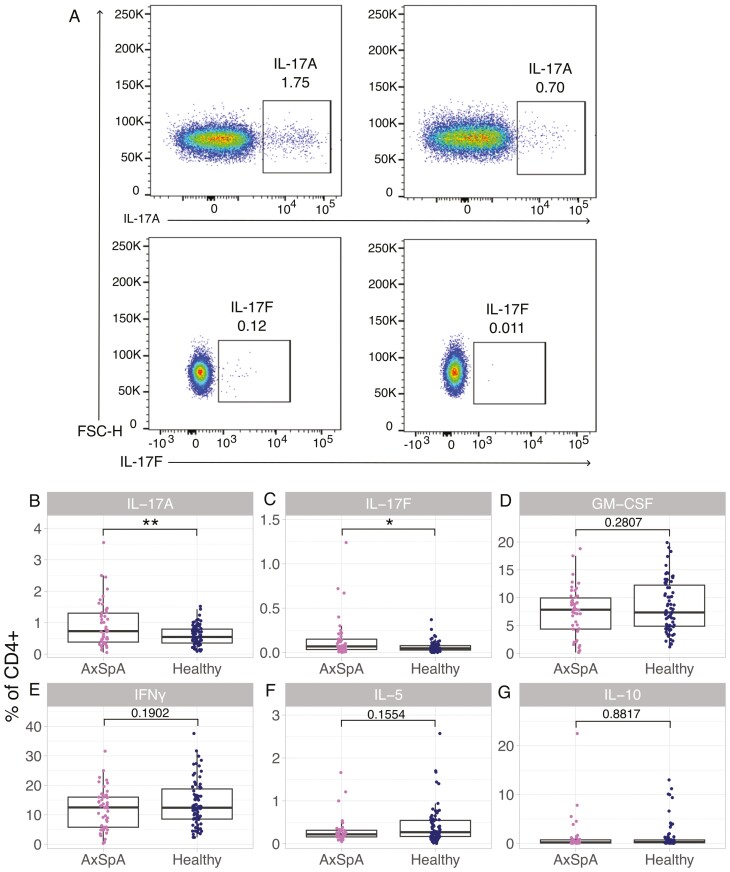
axSpA patients have increased frequencies of circulating IL-17A- and IL-17F-producing cells. PBMCs were isolated from 46 axSpA patients and 74 healthy controls and intracellular cytokine staining was completed on them. (**A**) Representative flow cytometry plots showing intracellular staining of IL-17A and IL-17F in axSpA and healthy donor PBMCs (gated on CD3+ CD4+ lymphocytes, detailed gating strategy is shown in Supplementary Fig. 9). (**B**–**G**) Frequencies of (B) IL-17A+, (**C**) IL-17F+, (**D**) GM-CSF+, (**E**) IFNγ+, (**F**) IL-5+, (**G**) IL-10 + T helper cells. **P < 0.01, *P < 0.05. Unpaired student’s *t*-test with Welch’s correction. Boxplots denote the median + interquartile range and individual data points are shown

We next sought to determine whether the elevation of cytokines in the axSpA stool was associated with the production of type-17 cytokines from circulating T-helper cells, which have previously been shown to be increased in axSpA [14]. We performed intracellular cytokine staining on *ex vivo* PBMCs from 46 patients with axSpA and 74 age and sex-matched healthy donors (Supplementary Table 1). We identified a range of cytokines produced by different subsets of T cells, including IL-17A, IL17F, and GM-CSF production by Th17 cells, IFNγ production by Th1 cells, IL-5 production from Th2 cells, and IL-10 production from T-regulatory cells [14, 21]. As described in previous studies [14], IL-17A+ CD4+ (Th17) cells were significantly increased in axSpA patients compared to healthy donors (Fig. 3A and B). IL-17F+ CD4+ cells were also significantly increased in patients with axSpA (Fig. 3A and C). We did not observe any significant differences in CD4+ producing GM-CSF, IFNγ, IL-5, or IL-10 cells between axSpA patients and healthy donors in this cohort (Fig. 3D–G).

To determine whether type-17 cytokines in the stool were associated with their production by circulating lymphocytes, we correlated stool cytokines and cytokine production by CD4+ T cells. We did not observe a correlation between circulating IL-17A- or GM-CSF-producing lymphocytes, and stool IL-17A or GM-CSF levels, respectively (Supplementary Fig. 7A and C). We aimed to validate this finding using surface markers CCR6 and CD161 found on Th17 cells. We found that CCR6+ CD161+ T helper cells correlated with IL-17A but not IL-17F production from circulating CD4+ cells, and not with stool IL-17A levels (Supplementary Fig. 7E–G). We calculated the integrated mean fluorescence intensity (iMFI) of each cytokine as it is a more accurate readout for cytokine production compared to the frequency of cytokine-producing cells [22]. However, the iMFI also did not correlate with stool cytokine levels (Supplementary Fig. 7B and D).

HLA-B27 is strongly associated with axSpA, and HLA-B27-transgenic rats display an expansion in IL-17A-producing CD4+ T cells [23]. Therefore, we stratified axSpA patients and healthy controls based on HLA-B27 status to determine whether HLA-B27 presence was associated with blood T-cell production of type-17 cytokines and their levels in the stool. However, we found that HLA-B27 was not associated with stool protein levels or cytokine-producing T cells, though a larger cohort study is needed to validate this (Supplementary Fig. 8A–K). We also found that the exclusion of axSpA patients with negative or unknown HLA-B27 status did not alter the correlation of stool proteins and circulating production of cytokines (Supplementary Fig. 8L–N).

## Discussion

We here describe for the first time the elevation of three type-17 cytokines (IL-17A, IL-23, and GM-CSF) in axSpA stool. Stool cytokine detection has been previously studied in the context of COVID-19, cirrhosis, and IBD; identifying elevated stool TNFα in IBD, but not inflammatory arthritis [13, 24–26]. The ability to use non-invasive stool cytokine detection in axSpA may allow for the characterization of the intestinal immune response without the need for intestinal biopsies. Indeed, the IL-23/IL-17 pathway has been strongly associated with axSpA, indicated by the clinical efficacy of IL-17 inhibition treatment, and prophylactic blockade of IL-23 inhibiting disease development in animal models of axSpA [14, 27, 28]. Our data suggest heightened local type-17 intestinal immunity in axSpA, in line with previous studies demonstrating elevated intestinal expression of IL-23 in axSpA and expansion of intestinal IL-17-producing innate lymphoid cells [29, 30]. However, *in vivo*, animal studies validating the use of stool cytokine detection in predicting intestinal immune responses and inflammation should be completed to strengthen this methodology. Furthermore, future normalization of stool cytokine assays to stool protein levels rather than total stool weight may allow for more detection accuracy due to the consideration of fibre and water content.

We demonstrated a negative correlation of stool IL-23 with multiple stool amino acid levels, potentially linking the function of the gut microbiome with local type-17 responses, although this does not indicate causality. Our cohort of axSpA patients also demonstrated increased levels of stool calprotectin, despite additional exclusion of diagnosed IBD (present in 7/53 of the axSpA patients studied). This finding was expected and consistent with the known association of axSpA with IBD and the presence of subclinical intestinal inflammation in up to 70% of patients [2]. We observed a positive correlation of stool calprotectin with stool IL-23 levels, though only when both patients and controls were included. This finding must therefore be regarded as requiring replication in a larger independent cohort of patients and controls. Our data are consistent with previous studies that have demonstrated a level of low-grade intestinal inflammation in axSpA, but now link this specifically to local gut type-17 inflammation. These findings further strengthen the gut–joint connection in axSpA, provide support for the concept that local intestinal type-17 responses may be pathogenic in SpA, and reinforce the efficacy of IL-17 inhibition in axSpA [28, 31]. We found that stool cytokine levels did not correlate with circulating production of cytokines, or surface markers for Th17 cells. This may be due to the production of type-17 cytokines by other immune cell subsets such as ILCs or maybe that intestinal and peripheral production of cytokines are regulated independently of one another [29]. Therefore, future mechanistic studies should interrogate the regulation of intestinal and peripheral cytokine production in axSpA.

Detection of cytokines in the stool raises the question as to whether these translocate directly through the intestinal epithelial layer or accompany the shedding of host type-17 cytokine-producing cells into the intestinal lumen. In order to address this question, we measured three markers of intestinal permeability in the serum. We were unable to detect changes in these markers, unlike a previous study [32]. Further larger studies of axSpA patients and controls, including functional measures of intestinal permeability such as the lactulose:mannose test are required to definitively determine the nature of intestinal permeability changes in axSpA.

We found that concentrations of several essential and nonessential amino acids in axSpA stool were negatively correlated with stool IL-23. One possible hypothesis is that bacterial metabolism of intestinal amino acids is associated with enhanced local intestinal type-17 responses. Previously published data has shown that leucine metabolism plays a critical role in T-cell activation, and that aspartate can dictate macrophage polarization [33, 34]. IL-23 production may also shape the intestinal microbiome and influence bacterial metabolism by promoting an inflammatory environment. For instance, IL-23 drives antimicrobial peptide production from intestinal epithelial cells, which can influence the intestinal microbiome [35]. In this regard, depletion of nonessential amino acids is also associated with intestinal barrier breakdown [36]. Overall, the potential association of stool cytokines with microbial metabolic function identified in our study support the use of stool cytokine detection alongside treatments modulating the microbiome including antibiotics and probiotics. This would allow for a noninvasive measure of the impact of manipulating the microbiome on the host immune response. However, our study design cannot show causation and further functional studies are required in order to mechanistically dissect the role of intestinal microbiome and metabolites in axSpA.

In summary, our study shows evidence of increased type-17 cytokines within the gut lumen in axSpA. These support a model in which gastrointestinal-generated type-17 immunity may contribute to pathogenesis, though a larger cohort study should be completed to replicate these findings. Our data suggest the potential use of stool biomarkers in axSpA diagnosis and the potential for treating axSpA by using gut-directed therapy to restore type-17 homeostasis. In this regard, future longitudinal studies measuring stool cytokine levels in axSpA patients alongside biologic treatment would allow for the assessment of stool cytokines as a biomarker for disease activity or for predicting response to treatment.

## Supplementary Material

kyae005_suppl_Supplementary_Materials

## Data Availability

The data underlying this article will be shared on reasonable request to the corresponding author.
