# Supplementary material for: Elevated type-17 cytokines are present in axial spondyloarthritis stool
Source: Discov Immunol. 2024 May 4;3(1):kyae005. doi: 10.1093/discim/kyae005 (PMC11222980; doi:10.1093/discim/kyae005)
Supplement: kyae005_suppl_Supplementary_Materials [file kyae005_suppl_supplementary_materials.docx]

# Supplementary Materials

|  | **AxSpA** | **Healthy Controls** |
| --- | --- | --- |
| **Number collected** | 53 | 91 |
| **Age, mean (range)** | 50.92 (24-74) | 52.14 (24-68) |
| **Sex, male/female** | 40/13 | 42/49 |
| **HLA-B27 -/unknown/+** | 6/4/43 | 39/12/40 |
| **Bath Ankylosing Spondylitis Disease Activity Index (BASDAI), mean (range)** | 3.91 (0.6-8.4) | N/A |
| **C-reactive protein (CRP), mean (range)** | 5.13 (0.2-31.9) | Not assessed |
| **Disease duration (years), mean (range)** | 16.95 (0.77-56.96) | N/A |
| **Non-steroidal anti-inflammatory drug (NSAID) usage** | 32 | 12 |
| **Biologic therapy, aTNF/aIL17** | 17/2 | N/A |
| **Disease modifying antirheumatic drug (DMARD) therapy** | 7 | N/A |
| **IBD, present/absent** | 7/46 | 0/91 |
| **Psoriasis, present/absent** | 9/44 | 0/91 |
| **Uveitis, present/absent** | 18/35 | 0/91 |
| **Enthesitis, present/absent** | 7/46 | 0/91 |
| **Dactylitis, present/absent** | 3/50 | 0/91 |

**Supplementary Table 1** Patient and control demographics.

| **Protein** | **Supplier** |
| --- | --- |
| Calprotectin (S100A8/9) | BioLegend |
| GM-CSF | Sigma-Aldrich |
| IFNγ | Sigma-Aldrich |
| IL-5 | R&D Systems |
| IL-6 | Sigma-Aldrich |
| IL-10 | Sigma-Aldrich |
| IL-17A | Sigma-Aldrich |
| IL-17F | R&D Systems |
| IL-21 | Sigma-Aldrich |
| IL-22 | Sigma-Aldrich |
| IL-23 | Sigma-Aldrich |
| Oncostatin M (OSM) | R&D Systems |
| TNFα | Sigma-Aldrich |

**Supplementary Table 2** Proteins quantified by stool ELISA on patients with axSpA and healthy controls.

| **Marker** | **Flourophore** | **Clone** | **Supplier** |
| --- | --- | --- | --- |
| CD3 | BV786 | OKT3 | BioLegend |
| CD4 | BV510 | RPA-T4 | BioLegend |
| CD8 | PerCP/Cy5.5 | RPA-T8 | BioLegend |
| Fixable viability dye | eFluor780 | N/A | ThermoFisher |
| IL-10 | BV711 | JES3-9D7 | BD Biosciences |
| IL-17A | BV650 | N49-653 | BD Biosciences |
| IL-17F | PE | O33-782 | BD Biosciences |
| IFNγ | AF700 | B27 | BioLegend |
| IL-5 | APC | TRFK5 | BD Biosciences |
| GM-CSF | PE-CF594 | BVD2-21C11 | BD Biosciences |
| CCR6 | BV510 | 29-2L17 | BioLegend |
| CD161 | PE/Dazzle 594 | HP-3G16 | BioLegend |

**Supplementary Table 3** Antibodies used for staining of PBMCs.

**Supplementary Figure 1 Th17-associated stool cytokines correlate with one another.** Correlation analyses were completed between stool cytokines. Pink points refer to patients with axSpA, purple points refer to healthy controls. A) Stool IL-23 correlated against stool IL-17A. B) Stool IL-17A correlated against stool GM-CSF. C) Stool IL-23 correlated against stool GM-CSF. D) Stool IL-10 correlated against stool IL-17A. E) Stool IL-10 correlated against stool GM-CSF. F) Stool IL-23 correlated against stool IL-10. Stool cytokine levels are denoted as pg/g of stool. Pearson’s correlation was carried out with Benjamini-Hochberg adjustment for multiple testing. P-value is denoted on each plot.

**Supplementary Figure 2 Patients with axSpA display elevated stool calprotectin even in the absence of diagnosed IBD.** A) Stool calprotectin (ng/g of stool) in axSpA patients (excluding those with an IBD diagnosis) compared to healthy controls. B-E) Stool protein levels in axSpA patients with diagnosed IBD compared to those without diagnosed IBD. B) Stool calprotectin (ng/g of stool). C) Stool IL-17A (pg/g of stool). D) Stool IL-23 (pg/g of stool). E) Stool GM-CSF (pg/g of stool). **p<0.01. Unpaired student’s t-test with Welch’s correction. Dotplots denote mean + standard error and individual data points are shown.

**Supplementary Figure 3 Stool calprotectin positively correlates with stool IL-23 levels.** Correlation analyses were completed between stool calprotectin and other immune parameters. Pink points refer to patients with axSpA, purple points refer to healthy controls. A) Stool IL-23 (pg/g of stool) of pooled axSpA and healthy controls (p=0.0023, r=0.2848). B) Stool IL-23 (pg/g of stool) of only axSpA patients (p=0.235, r=0.2283). Pearson’s correlation was carried out with Benjamini-Hochberg adjustment for multiple testing. P-value is denoted on each plot.

**Supplementary Figure 4 Disease activity measures and disease duration do not correlate with significant effects on stool calprotectin or type-17 cytokine production**. A) Stool calprotectin (ng/g of stool), B) stool IL-17A (pg/g of stool), C) stool IL-23 (pg/g of stool), D) stool GM-CSF (pg/g of stool), E) IL-17A+ (% of CD4+), F) IL-17F+ (% of CD4+). Pearson’s correlation was carried out with Benjamini-Hochberg adjustment for multiple testing. P-value is denoted on each plot.

**Supplementary Figure 4 (continued from previous page).** G) IL-5+ (% of CD4+), H) IL-10+ (% of CD4+), I) GM-CSF+ (% of CD4+), J) IFNγ+ (% of CD4+). Pearson’s correlation was carried out with Benjamini-Hochberg adjustment for multiple testing. P-value is denoted on each plot.

**Supplementary Figure 5 AxSpA NSAID and biologic usage do not associate to significant effect with stool calprotectin or type-17 cytokine production**. A) Stool calprotectin (ng/g of stool), B) stool IL-17A (pg/g of stool), C) stool IL-23 (pg/g of stool), D) stool GM-CSF (pg/g of stool), E) IL-17A+ (% of CD4+), F) IL-17F+ (% of CD4+), G) IL-5+ (% of CD4+), H) IL-10+ (% of CD4+), I) GM-CSF+ (% of CD4+), J) IFNγ+ (% of CD4+). Unpaired student’s t-test with Welch’s correction and Benjamini-Hochberg adjustment for multiple testing. Dotplots denote mean + standard error and individual data points are shown.

**Supplementary Figure 6 I-FABP, LBP, and s-CD14 (proteins indicative of intestinal barrier function) are not significantly altered in AxSpA.** Serum was collected from 77 axSpA patients and 84 healthy controls, and intestinal fatty acid binding protein (I-FABP), LPS binding protein (LBP), and soluble (s)-CD14 were quantified by ELISA. A) Serum I-FABP, B) serum LBP, C) serum s-CD14. Protein levels are denoted as pg/mL of sera. Unpaired student’s t-test with Welch’s correction. Boxplots denote median + interquartile range and individual data points are shown.

**Supplementary Figure 7 Stool cytokines do not correlate to significant effect with peripheral blood CD4+ T cell cytokine production.** A-D) Correlation analyses were performed between stool cytokine levels and both frequencies and integrated MFI values of cytokines produced by circulating CD4+ T cells. A) IL-17A+ (% of CD4+) and stool IL-17A (pg/g of stool). B) IL-17A+ (iMFI) and stool IL-17A (pg/g of stool). C) GM-CSF+ (% of CD4+) and stool GM-CSF (pg/g of stool). D) GM-CSF (iMFI) and stool GM-CSF (pg/g of stool). E-G) CD4+ T cells displaying surface markers (CCR6 and CD161) for Th17 cells were correlated against stool IL-17A and circulating production of IL-17A and IL-17F by CD4+ T cells. E) Stool IL-17A (pg/g of stool) and CCR6+ CD161+ (% of CD4+). F) CCR6+ CD161+ (% of CD4+) and IL17A+ (% of CD4+). G) CCR6+ CD161+ (% of CD4+) and IL17F+ (% of CD4+). Pink points refer to patients with axSpA, purple points refer to healthy controls. Pearson’s correlation was carried out with Benjamini-Hochberg adjustment for multiple testing. P-values are denoted on each plot.

**Supplementary Figure 8 HLA-B27 status is not associated to a significant effect with stool cytokine levels or circulating production of cytokines.** A-K) stool protein levels and circulating cytokine production by CD4+ T cells from axSpA patients and healthy controls were plotted based upon HLA-B27 positivity. A) Stool calprotectin (ng/g of stool). B) Stool IL-17A (pg/g of stool). C) Stool IL-23 (pg/g of stool). D) Stool GM-CSF (pg/g of stool). E) Stool IL-10 (pg/g of stool). F) IL-17A+ (% of CD4+). G) IL-17F+ (% of CD4+). H) GM-CSF (% of CD4+). I) IFNγ+ (% of CD4+). J) IL-10+ (% of CD4+). K) IL-5+ (% of CD4+). One-way ANOVA with Tukey’s post-hoc testing was completed for all plots. ****p<0.0001, ***p<0.001, **p<0.01, *p<0.05. L-N) Correlation analyses of stool proteins and circulating cytokine production was completed with axSpA patients including (top graphs) and excluding (bottom graphs) those with unknown or negative HLA-B27 status. L) Stool IL-23 (pg/g of stool) and stool calprotectin (ng/g of stool). M) IL-17A+ (% of CD4+) and stool IL-17A (pg/g of stool). N) GM-CSF+ (% of CD4+) and stool GM-CSF (pg/g of stool). Pearson’s correlation was carried out with Benjamini-Hochberg adjustment for multiple testing. P-values are denoted on each plot.

**Supplementary Figure 9 Gating strategy for intracellular flow cytometry.** Gating strategy for cytokine staining of GM-CSF, IL-17F, IFNɣ, IL-10, IL-5, and IL-17A+ production from PBMCs.
